# ECMO management for severe pulmonary embolism with concurrent cerebral hemorrhage: a case report

**DOI:** 10.3389/fcvm.2024.1410134

**Published:** 2024-05-13

**Authors:** Lutao Xie, Pin Lan, Mingjun Liu, Kechun Zhou

**Affiliations:** Department of Emergency, Lishui Central Hospital, Zhejiang, China

**Keywords:** pulmonary embolism, ECMO, cerebral hemorrhage, anticoagulation, balloon pulmonary angioplasty

## Abstract

**Background:**

Acute pulmonary embolism (APE) is a common and potentially fatal cardiovascular disease that can lead to sudden cardiac arrest in severe cases. When conventional cardiopulmonary resuscitation measures fail to achieve the return of spontaneous circulation (ROSC) in patients with APE, venoarterial extracorporeal membrane oxygenation (ECMO) becomes a viable therapeutic option. As an advanced life support treatment, ECMO ensures the perfusion of critical organs, providing sufficient time for interventions necessary for ROSC.

**Case introduction:**

We report the case of a patient who experienced cardiac arrest due to pulmonary embolism. During the treatment, the patient received two sessions of external cardiopulmonary resuscitation (ECPR) as supportive care and experienced cerebral hemorrhage. Ultimately, the patient improved and was discharged following support from extracorporeal membrane oxygenation (ECMO), careful anticoagulation strategies, and intervention with balloon pulmonary angioplasty.

**Conclusion:**

ECMO can serve as an important life support technology for patients with severe APE. Through a cautious anticoagulation therapy, not only was the ECMO support successfully maintained but also was further deterioration of cerebral hemorrhage effectively prevented. For patients with concurrent main pulmonary artery embolism and bleeding, balloon pulmonary angioplasty may be an option.

## Introduction

Epidemiological studies indicate that pulmonary embolism (PE) is the third most common cause of sudden death in adults, closely following heart diseases and stroke. Annually, an epidemiological study suggests that the incidence rate of PE is between 39 and 115 cases per 100,000 people (1). Despite the increasing availability of diagnostic methods and medical and surgical treatment options, the mortality rate for acute pulmonary embolism (APE) remains high: up to 70% in patients with hemodynamic instability or circulatory shock within the first hour (2). The main standard treatment modalities for APE include anticoagulation, thrombolysis, catheter-based, and surgical interventions (3). For patients with severe APE and cardiac arrest in whom conventional cardiopulmonary resuscitation measures fail to achieve a return of spontaneous circulation (ROSC), extracorporeal membrane oxygenation (ECMO) can provide organ perfusion, allowing sufficient time for interventions necessary for ROSC (4). For patients with concurrent main pulmonary artery embolism and bleeding, anticoagulation management during ECMO treatment poses a greater challenge. In our report, we describe and discuss the case of a patient with severe PE and cerebral hemorrhage who was successfully treated with life support via ECMO, individualized anticoagulation therapy, and catheter-based intervention.

## Case report

**Figure 1:**
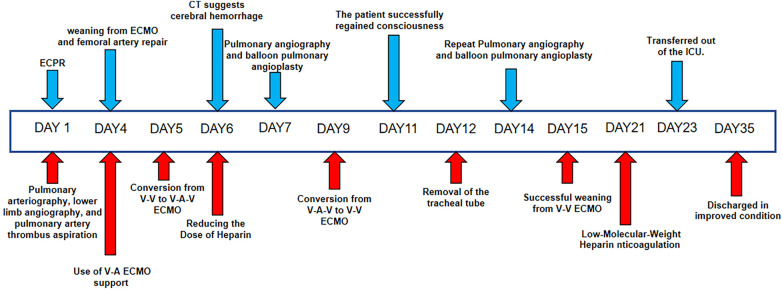
Patient's treatment course.

**Figure 2:**
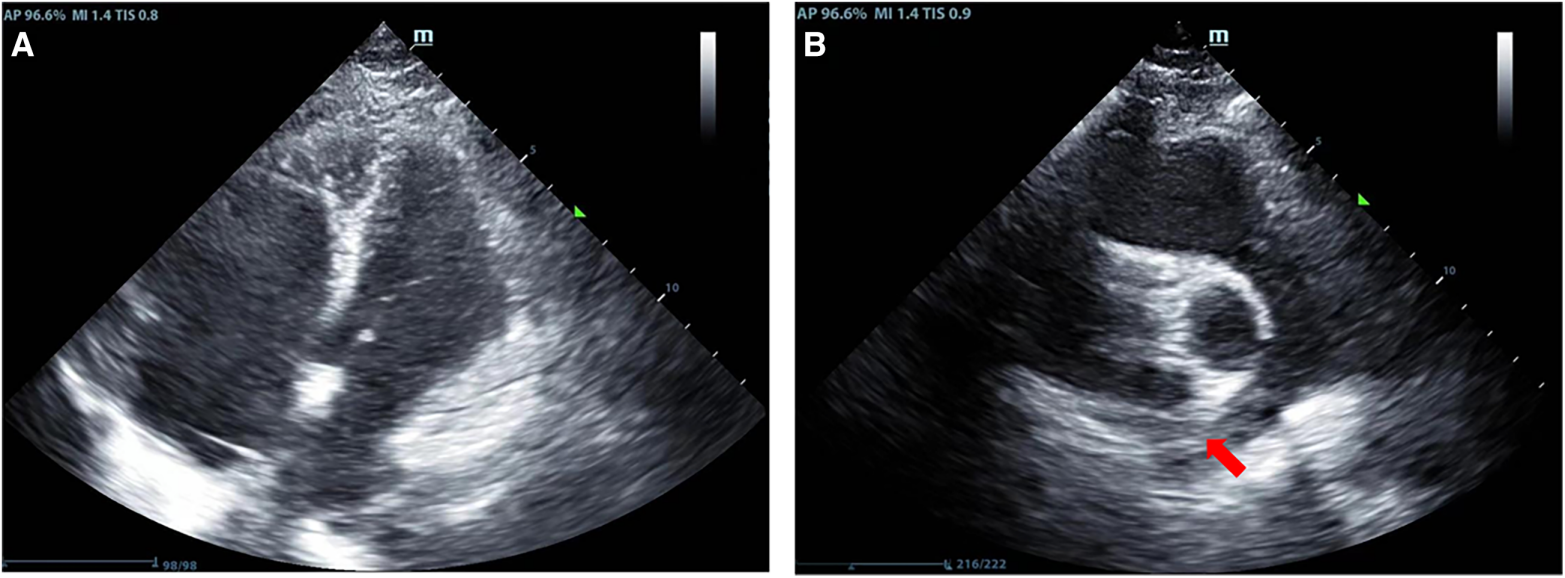
Results of the patient's echocardiography. (**A**) Indicates enlargement of the right heart in the patient; (**B**) Shows thrombus echoes in the main pulmonary artery of the right lung (red arrow).

**Figure 3:**
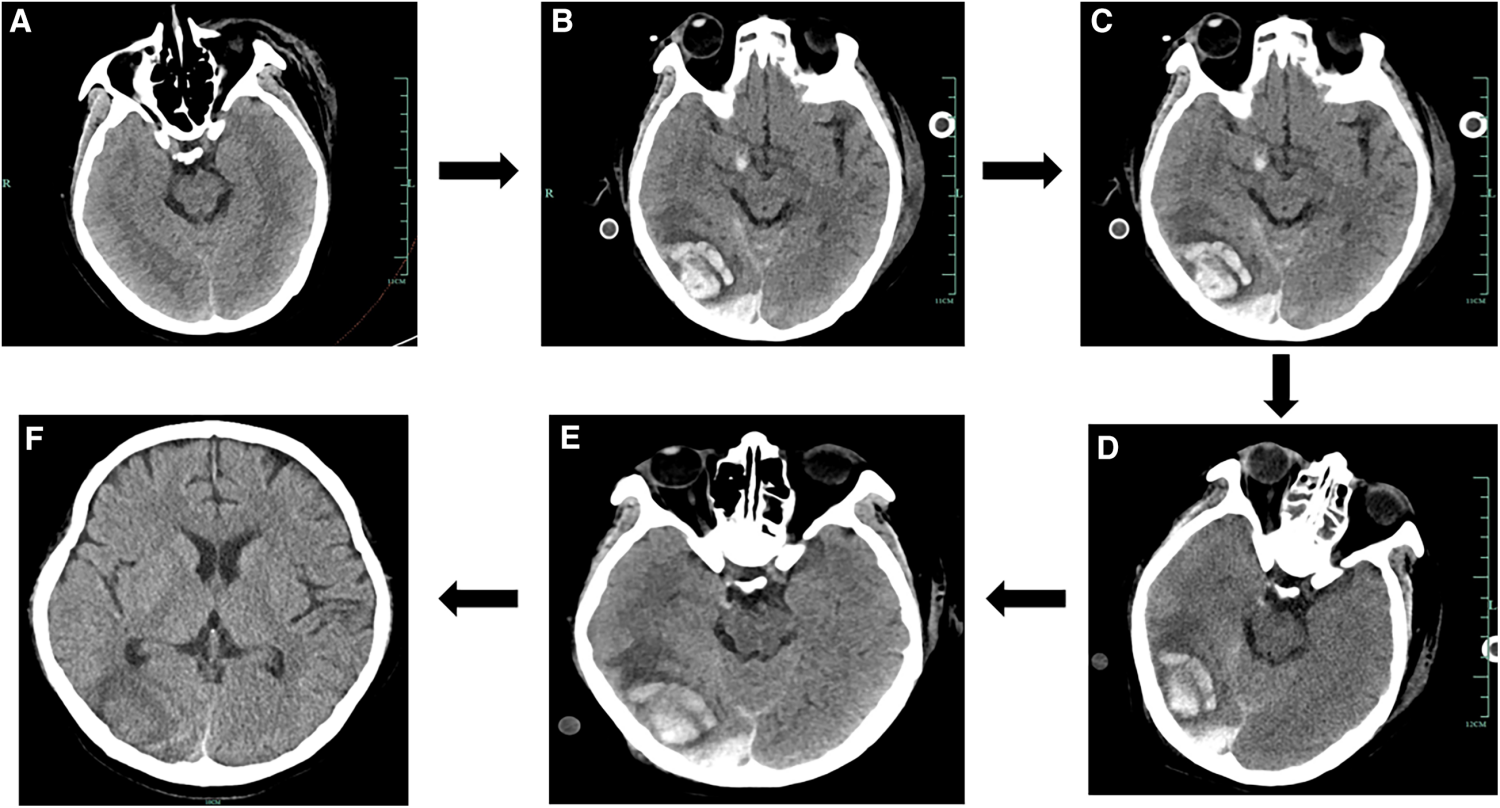
Results of the cranial CT scans during the patient's hospital stay. (**A**) Results of the patient's cranial CT on Day 1. (**B**) Results of the patient's cranial CT on Day 6. (**C**) Results of the patient's cranial CT on Day 9. (**D**) Results of the patient's cranial CT on Day 11. (**E**) Results of the patient's cranial CT on Day 15. (**F**) Results of the patient's cranial CT on Day 35.

**Figure 4:**
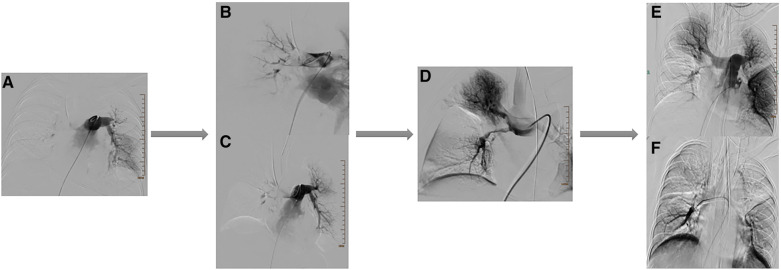
Results of multiple angiographies during the patient's treatment period. (**A**) Images of the patient's pulmonary angiography. (**B**,**C**) Images after treatment of the patient's pulmonary embolism. (**D**) Images after the patient's first balloon pulmonary angioplasty (BPA) treatment. (**E**,**F**) Images after the patient's second balloon pulmonary angioplasty (BPA) treatment.

A previously healthy 43-year-old female patient was urgently brought by emergency services and admitted to the hospital after suddenly losing consciousness 20 min before admission because of a transient episode of syncope without any apparent trigger, which resulted in head trauma. En route to the hospital in an ambulance, the patient suffered a cardiopulmonary arrest, prompting emergency medical personnel to commence cardiopulmonary resuscitation (CPR) until reaching our emergency department. Figure 1 is a timeline of the clinical condition progress and major management of the patient. Our medical team, in addition to ongoing CPR efforts, rapidly performed tracheal intubation and assisted ventilation among other resuscitative measures. Due to the failure of conventional CPR, our medical team urgently initiated extracorporeal cardiopulmonary resuscitation (ECPR). Thirty-eight minutes post-admission, the patient successfully regained spontaneous cardiac rhythm under V-A ECMO support. Laboratory tests on admission were as follows (Before V-A ECMO initiation): complete blood count (white blood cells, 13.7 × 10^9^/L; hemoglobin, 144 g/L; platelets, 71 × 10^9^/L), blood gas analysis (Arterial blood) (pH, 6.748; CO_2_, partial pressure, 104.0 mmHg; oxygen partial pressure, 27.3 mmHg; total blood lactate level, 19.0 mmol/L; reduced hemoglobin, 84.8 g/L), biochemistry (alanine transaminase, 63 U/L; myoglobin, 106.9 ng/ml), and coagulation profile (international normalized ratio, 1.42; activity, 57%; fibrinogen, <0.70 g/L; D-dimer, 84.72 mg/L; fibrin degradation products, 276.21 μg/ml). Her brain natriuretic peptide and procalcitonin levels were 70.0 pg/ml and 0.04 ng/ml, respectively. Chest computed tomography angiography scan indicated bilateral PE. Echocardiogram findings (after V-A ECMO initiation) included right heart enlargement, thrombus echoes observed within the main pulmonary artery of the right lung (Figures 2A,B), with a left ventricular internal diameter in diastole (LVIDd) of 29.3 mm, left ventricular internal diameter in systole (LVIDs) of 26.4 mm, and a left ventricular ejection fraction (LVEF) of 22.8%. Cranial CT scan showed no evidence of intracranial hemorrhage (Figure 3A). Duplex ultrasonography of the lower extremities revealed a venous thrombus in the right leg. Cardiopulmonary arrest, PE, and lower limb venous thrombosis were suspected; hence, the admission. Our medical team deduced that PE was likely caused by the detachment of a lower limb venous thrombus and urgently performed pulmonary angiography and thrombectomy. The procedure revealed the thrombus to be hard in texture (considered white thrombus), with multiple attempts at aspiration and excision achieving suboptimal results, yet successfully restoring blood flow in the apical and anterior segments of the left pulmonary artery and the right upper pulmonary artery (Figures 4A–C). Postoperatively, the dosage of unfractionated heparin was adjusted via an infusion pump, keeping the partial thromboplastin time between 50 s and 70 s. In the subsequent treatment, the use of vasopressor medications gradually decreased, cardiac ejection fraction improved, and oxygen saturation levels increased. After comprehensive assessment, ECMO decannulation, femoral artery repair, and ligation of the right great saphenous vein were performed on the fourth day of hospitalization. However, 3 h postoperatively, the patient's oxygen saturation levels began to decrease, associated with shock, leading to another episode of cardiac arrest; hence, the V-A ECMO support was reactivated. Analysis suggested that the obstruction in the right pulmonary artery's main trunk severely impacted the patient's oxygen partial pressure and blood pressure. Considering the patient's current hypotension and hypoxemia, our medical team adjusted the ECMO mode from V-A to V-A-V on the fifth day of hospitalization. On the sixth day, under ECMO support, the patient completed a head, chest, and abdominal CT assessment, unexpectedly revealing cerebral hemorrhage (Figure 3B), thought to likely result from the combined effects of pre-CPR fall history and heparin anticoagulation; the presence of an edema halo around the hemorrhage suggested the bleeding had been present for several days and was relatively stable. Following multidisciplinary consultation, the anticoagulation treatment was adjusted, aiming to keep the partial thromboplastin time at 40–50 s. Due to suboptimal outcomes from pulmonary artery thrombectomy for the thrombus in the patient's right pulmonary artery main trunk and considering the current cerebral hemorrhage which could not withstand surgery requiring high doses of anticoagulants, a multidisciplinary decision was made to proceed with balloon pulmonary angioplasty (BPA) for the patient. On the seventh day of hospitalization, the patient underwent pulmonary angiography and balloon pulmonary angioplasty, with wire-guided balloon dilation restoring blood flow in the basal segment of the right lower lobe (Figure 4D). After continuous assessment and treatment, the patient's condition further stabilized. On the ninth day, a follow-up cranial CT scan reassuringly showed no significant increase in intracranial hemorrhage (Figure 3C). With significant improvement in cardiac function and effective reduction in pulmonary artery pressure, our team adjusted the ECMO mode from V-A-V to V-V. On the 11th day, after confirming no increase in intracranial hemorrhage via cranial CT scan (Figure 3D), our team decided to wean the patient off sedation and initiate ventilator weaning exercises; the patient successfully awoke and was able to follow commands. By the 12th day, while continuing on V-V ECMO support, the patient was extubated and introduced to music therapy. Our team monitored changes in the patient's consciousness and muscle strength, indirectly assessing the intracranial hemorrhage status. To shorten the duration of ECMO treatment, on the 14th day of hospitalization, our medical team decided to perform pulmonary angiography and balloon pulmonary angioplasty again and precisely dilated the balloon in the basal and upper arterial segments of the patient's right lung to enhance ventilation-perfusion matching (Figures 4E,F). Eventually, on the 15th day of hospitalization, the patient's oxygen saturation significantly improved, pulmonary artery pressure dropped from 49 mmHg to 28 mmHg (as measured by bedside ultrasound), and after multiple weaning assessments, V-V ECMO was successfully removed. The medical team closely monitored the patient's intracranial hemorrhage condition (Figure 3E) until on the 21st day of hospitalization, the patient was switched to low-molecular-weight heparin anticoagulation therapy (0.4 ml q12 h). After a series of treatments and care, the patient was successfully transferred to a general ward on the 23rd day of hospitalization. Ultimately, the patient was discharged in improved condition after 35 days of treatment, with follow-up cranial CT scans showing the hemorrhage had essentially been absorbed (Figure 3F).

## Discussion

Several studies have confirmed that ECMO can serve as an effective adjunctive treatment for patients highly at risk for PE (5, 6). Notably, during ECMO treatment, patients face a higher risk of bleeding, with the occurrence of cerebral hemorrhage potentially severely impacting the prognosis. It has been reported that about 37% of patients may experience complications such as cerebral hemorrhage or stroke (7). In this case, the patient had a history of head trauma at the onset of the condition, combined with systemic heparin anticoagulation during ECMO operation, which ultimately led to cerebral hemorrhage. Despite the absence of specific anticoagulation guidelines for ECMO operation combined with cerebral hemorrhage, studies have shown that V-V ECMO treatment in patients with cranial trauma, with proper anticoagulation, does not necessarily lead to increased intracranial hemorrhage (8, 9). Based on relevant literature, we ultimately chose to maintain the activated partial thromboplastin time within the target range of 40–50 s, reducing the risk of increased intracranial hemorrhage during treatment, thus maximizing patient safety and treatment efficacy (10, 11). During this period, we assessed the patient's coagulation and platelet functions by analyzing thromboelastography, which aided in guiding our anticoagulation strategy.

According to the 2019 European Society of Cardiology Guidelines for the diagnosis and management of APE developed in collaboration with the European Respiratory Society, in cases of refractory circulatory failure or cardiac arrest, the use of ECMO combined with surgical embolectomy or catheter-directed treatment may be considered (12). In this case, large- and small-bore embolectomy and rheolytic thrombectomy surgical plans are based on the latest interventional treatment strategies at the beginning of treatment (13). However, due to the massive thrombus in the patient's right pulmonary artery (considered to be a white thrombus), the interventional treatment was not ideal. Postoperatively, only a heparin anticoagulation strategy was adopted without performing surgical embolectomy, which likely led to our first ECMO weaning failure. Surgical embolectomy is often used in cases where other treatments have failed or are contraindicated, considering that the patient's PE was caused by the detachment of a lower limb venous thrombus, and ECMO combined with surgical embolectomy might have been the most appropriate treatment for the patient (14). During the second ECMO treatment period, the patient developed cerebral hemorrhage, making it impossible to tolerate surgical embolectomy; hence, multiple BPA treatments were decided. Balloon pulmonary angioplasty is a surgery used to treat chronic thromboembolic pulmonary hypertension, often used as an interventional treatment method for patients who cannot tolerate open surgery (15). This method has been widely applied for the treatment of chronic thromboembolic pulmonary hypertension; however, the application of BPA for the treatment of APE has not been mentioned. With two BPA treatments and the transition from V-V to V-A-V to V-V ECM, the patient was successfully weaned off ECMO and transferred out of the ICU, demonstrating that BPA might become an effective alternative treatment option for APE.

## Conclusion

ECPR is an effective adjunctive treatment for severe pulmonary artery embolism. During ECMO maintenance treatment, when severe cerebral hemorrhage complications arise, a cautious anticoagulation strategy can ensure treatment efficacy while ensuring patient safety. In patients with severe pulmonary artery embolism in whom conventional treatment is ineffective, BPA may become an effective alternative treatment option.

## Data Availability

The original contributions presented in the study are included in the article/Supplementary Material, further inquiries can be directed to the corresponding author.
